# The non-homologous end-joining pathway is involved in stable transformation in rice

**DOI:** 10.3389/fpls.2014.00560

**Published:** 2014-10-17

**Authors:** Hiroaki Saika, Ayako Nishizawa-Yokoi, Seiichi Toki

**Affiliations:** ^1^Plant Genome Engineering Research Unit, Agrogenomics Research Center, National Institute of Agrobiological SciencesTsukuba, Japan; ^2^Kihara Institute for Biological Research, Yokohama City UniversityYokohama, Japan

**Keywords:** *Agrobacterium*, non-homologous end-joining, rice, stable transformation, T-DNA

## Abstract

Stable transformation with T-DNA needs the coordinated activities of many proteins derived from both host plant cells and *Agrobacterium*. In dicot plants, including *Arabidopsis*, it has been suggested that non-homologous end-joining (NHEJ)—one of the main DNA double-strand break repair pathways—is involved in the T-DNA integration step that is crucial to stable transformation. However, how this pathway is involved remains unclear as results with NHEJ mutants in *Arabidopsis* have given inconsistent results. Recently, a system for visualization of stable expression of genes located on T-DNA has been established in rice callus. Stable expression was shown to be reduced significantly in NHEJ knock-down rice calli, suggesting strongly that NHEJ is involved in *Agrobacterium*-mediated stable transformation in rice. Since rice transformation is now efficient and reproducible, rice is a good model plant in which to elucidate the molecular mechanisms of T-DNA integration.

## INTRODUCTION

*Agrobacterium tumefaciens* enables genetic transformation of many plant species via the movement of transferred-DNA (T-DNA) of Ti plasmids into the plant nucleus. Despite its use in both basic research and molecular breeding in several crops, many species and varieties are still recalcitrant to *Agrobacterium*-mediated transformation. To overcome this limitation requires not only optimization of cell and tissue culture conditions but also elucidation of the molecular mechanisms of all the events that occur during *Agrobacterium*-mediated transformation.

In the conventional *Agrobacterium*-mediated transformation system in rice, callus derived from the scutellum of mature seeds or immature embryos is generally used for *Agrobacterium* inoculation (Nishimura et al., 2006; Hiei and Komari, 2008). Since these tissues have a stereo architecture consisting of many cells, *Agrobacterium* can only infect cells on the surface, and, unlike protoplasts, it is quite difficult to isolate single cells from these tissues without any selection pressure. Indeed, there are far fewer transformed than non-transformed cells when *Agrobacterium*-inoculated primary calli derived from mature seeds are cultured without selection pressure in a conventional transformation system (Saika and Toki, 2009; Saika et al., 2011b). Therefore, transformed cells in which antibiotic- and herbicide-resistance genes located on T-DNA are present, but not expressed to a sufficient level, cannot be distinguished from non-transformed cells.

**FIGURE 1:**
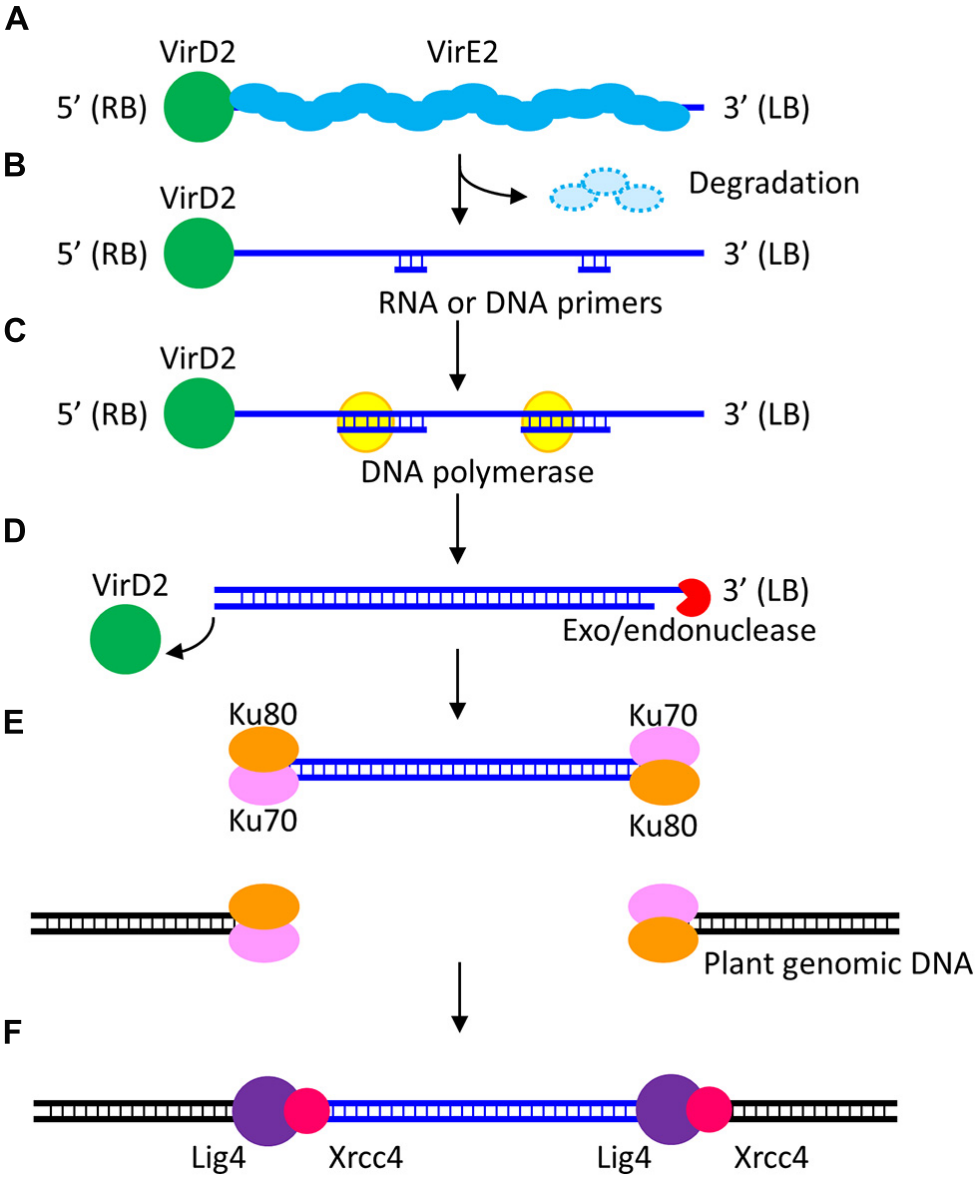
**A model of T-DNA integration into the host genome via the non-homologous end-joining (NHEJ) pathway.** A T-complex composed of ssT-DNA and virulence proteins VirD2 and VirE2 is transported to the host cell nucleus **(A)**. In the nucleus, VirE2 proteins, which bind the ssT-DNA, are removed by proteasomal degradation. Short DNA or RNA primers anneal randomly to the ssT-DNA **(B)** and then serve as primers for polymerization of the nascent T-DNA strand **(C)**. The 3′ end of the T-DNA is thought to be degraded by nucleases **(D)**. dsT-DNA molecules integrate into plant genomic double strand breaks (DSBs) via the NHEJ pathway. The Ku70/80 heterodimer binds and protects the DSB ends **(E)**. Lig4 is a ligase that joins the DSB ends. Xrcc4 interacts with Lig4 and enhances its activity **(F)**.

*Agrobacterium*-mediated transformation has many steps (Gelvin, 2010; Pitzschke and Hirt, 2010; Magori and Citovsky, 2011; Shiboleth and Tzfira, 2012). The last step, i.e., stable transgene expression that results from T-DNA integration into the host genome (and the avoidance of the gene silencing system) is crucial to the clonal propagation of transformed cells of rice as referred to above. Meanwhile, transient expression of transgenes is often observed at an earlier stage when transgenes are expressed stably and constantly from T-DNA that has not integrated into the rice genome. After import into the nucleus of the T-complex, which consists of single stranded T-DNA (ssT-DNA) and proteins such as virD2 and virE2, ssT-DNA is replicated to double-strand T-DNA (dsT-DNA). Hypothetical models of dsT-DNA formation have been proposed as shown in **Figure 1** (Liang and Tzfira, 2013). Subsequently, transgenes located on the dsT-DNA are expressed. However, this expression is not continuous because naked T-DNA that has not integrated into the host genome is susceptible to degradation (Gelvin, 2010). The transient expression of a cytokinin biosynthesis gene, which leaves no selection marker or vector backbone in the host genome, has been exploited in a transformation system for *Solanaceous* plants (Richael et al., 2008). However, it is thought that transient transgene expression is not crucial to the production of clonally propagated transformed cells in the case of rice.

## MOLECULAR MECHANISM OF STABLE TRANSFORMATION VIA *Agrobacterium* IN *Arabidopsis*

The molecular mechanisms of T-DNA integration into the plant genome in *Agrobacterium*-mediated transformation remain unclear. The sequences of T-DNA integration sites were determined in *Arabidopsis* and tobacco plants over 20 years ago (Gheysen et al., 1991; Mayerhofer et al., 1991). Based on these pioneer reports, two major models—the strand-invasion model and the double strand break (DSB) repair model—were proposed (Gelvin, 2010; Pitzschke and Hirt, 2010; Magori and Citovsky, 2011; Shiboleth and Tzfira, 2012). In the strand-invasion model, T-DNA integration is thought to be caused by microhomology-mediated repair between ssT-DNA and the plant genome. On the other hand, in the DSB repair model, dsT-DNA is thought to integrate via non-homologous end-joining (NHEJ)—one of the main DSB repair pathways—into the DSB sites that occur randomly in the plant genome (**Figure 1**).

Many proteins involved in the NHEJ pathway, such as Ku70, Ku80, Ligase 4 (Lig4), and Xrcc4, have been characterized in model plants such as *Arabidopsis* and rice (West et al., 2000; Riha et al., 2002; Kimura and Sakaguchi, 2006; Hong et al., 2010; Nishizawa-Yokoi et al., 2012). To summarize previous reports in *Arabidopsis*, on the whole, the frequency of stable transformation tends to decrease in NHEJ mutants except for *xrcc4*, although this point remains controversial. It was reported initially that the frequency of stable transformation increased twofold in *in planta* floral dip transformation experiments in *Arabidopsis ku80* mutants (Gallego et al., 2003), while another report demonstrated a two to threefold decrease (Friesner and Britt, 2003). However, a root tumorigenesis assay in *Arabidopsis ku80* mutants confirmed that the stable transformation frequency was decreased severely, although the frequency of transient transformation remained comparable to controls (Li et al., 2005b). In addition, overexpression of Ku80 enhanced stable but not transient transformation frequency (Li et al., 2005b). Moreover, recent reports have shown that stable transformation frequency was decreased two to fourfold in *Arabidopsis ku80* mutants in both root tumorigenesis assay and in *in planta* floral dip assay (Jia et al., 2012; Mestiri et al., 2014), and stable transformation frequency was also decreased fourfold in *Arabidopsis ku70* mutants in the *in planta* floral dip assay (Jia et al., 2012). Thus, *Arabidopsis* Ku70/80 plays a positive role in stable transformation. It was proposed that Ku80 proteins interact with dsT-DNA molecules and direct them to DSB sites (Li et al., 2005b). On the other hand, there are contrary reports in *Arabidopsis lig4* mutants: some authors found that stable transformation frequency was decreased to 40–70% in *in planta* floral dip assay (Friesner and Britt, 2003), but others found comparable rates to wild-type in both root tumorigenesis assay and *in planta* floral dip assay (van Attikum et al., 2003). These inconsistencies are probably due to differences in cell type (somatic cells vs germ cells) and/or experimental procedures (Gelvin, 2010; Nishizawa-Yokoi et al., 2012; Lacroix and Citovsky, 2013). Interestingly, the possibility that Xrcc4 plays a negative role in stable transformation via interaction with virE2 has been raised (Vaghchhipawala et al., 2012). In this latter theory, active Xrcc4 proteins are inhibited by virE2 proteins and T-DNA is integrated into DSB sites that are either not repaired or that occur *de novo* by suppression of the NHEJ pathway at a higher frequency. *Agrobacterium* might bring stable transformation to a successful conclusion by the good use of host proteins involved in the NHEJ pathway, among others Ku80 and Xrcc4. Just recently, Mestiri et al. (2014) reported that DSB repair pathways including NHEJ are redundantly involved in stable transformation. Stable transformation frequencies were decreased in the single mutants, *ku80*, *xrcc1*, *xrcc2*, and *xpf* (Mestiri et al., 2014), which are deficient in NHEJ, Ku-independent NHEJ (known as alternative NHEJ to distinguish it from classical Ku-dependent NHEJ), microhomology-mediated end joining/single strand annealing and homologous recombination, respectively. Interestingly, stable transformation was markedly but not completely suppressed in a quadruple mutant (Mestiri et al., 2014), possibly because the quadruple mutant did not lose DSB repair activity completely (Charbonnel et al., 2011).

## MOLECULAR MECHANISM OF STABLE TRANSFORMATION VIA *Agrobacterium* IN RICE

Unlike *Arabidopsis*, rice is not a natural host plant of *Agrobacterium*; however, the combination of a sophisticated tissue culture system and activation of *Agrobacterium* using the phenolic compound acetosyringon, allows transgenic rice plants to be produced via this method (Hiei et al., 1994). Human cells and yeast—also not natural hosts of *Agrobacterium*—are also susceptible to *Agrobacterium*-mediated transformation (Bundock et al., 1995; Kunik et al., 2001). Moreover, the NHEJ pathway is involved in T-DNA integration in yeast (van Attikum et al., 2001; van Attikum and Hooykaas, 2003). These findings suggest that *Agrobacterium* uses a fundamental DNA repair system that is common to both host and non-host organisms, including rice, in the step leading to stable transformation. Since no conventional root transformation system or *in planta* floral dip transformation system has been established in rice, we are currently restricted to rice callus for analyzing stable transformation frequencies. Tumorigenesis assays are not applicable to rice callus because this tissue has already dedifferentiated. In *Arabidopsis* and *Nicotiana*, stable transformation frequencies are estimated by visualization of transformed cells (Mysore et al., 1998; Anand et al., 2007). In rice, transient and stable transgene expression can also be observed using green fluorescent protein—a non-destructive and visible selection marker (Toki et al., 2006; Saika et al., 2012). Visualization of transformed cells can quantify stable transformation events rapidly and easily compared to clonal propagation of transformed cells using antibiotics. We have established a sequential monitoring system for stable transformation events in rice callus that uses the enhancer trap of the click beetle luciferase gene, which is 30 times more sensitive than firefly luciferase (Saika et al., 2012). Using this system, we showed that the stable transformation frequency is decreased in knock-down lines of *OsKu70*, *OsKu80,* and *OsLig4* genes and the knock-out line of the *OsKu70* gene (Nishizawa-Yokoi et al., 2012; Saika et al., 2012), suggesting that the NHEJ pathway is involved in the stable transformation process also in rice.

## PROSPECTS

According to the DSB repair model, DSBs could occur in cells successfully transformed with T-DNA. In *Arabidopsis*, DSBs induce cell death and endocycle, which halts cell division (Adachi et al., 2011). Cells in which these events occur cannot propagate clonally if stable transformation is successful. Thus, transformation frequency in *Arabidopsis* might be underestimated in mutants in which DSBs occurs at higher frequency, such as NHEJ deficient mutants. However, in rice callus, endocycle does not occur even under genotoxic conditions inducing DSBs (Endo et al., 2012). Thus, transformation frequency may be estimated more accurately in rice callus than in *Arabidopsis*.

Transformation frequency in *Arabidopsis* and rice depends on the variety (Nam et al., 1997; Saika and Toki, 2010). Furthermore, optimal conditions for cell and tissue culture differ among rice varieties (Hiei and Komari, 2008). For example, Koshihikari—an elite variety from Japan—shows lower regeneration frequency due to lower activity of nitrite reductase (Nishimura et al., 2005; Ozawa and Kawahigashi, 2006). This makes it difficult to compare transformation frequency precisely among rice varieties since different callus culture conditions can affect *Agrobacterium* activity and growth. Thus, in order to compare transformation frequency precisely, experiments must be performed under reliable, reproducible and identical conditions using mutants with the same genetic background. Recent technological advances have made it much easier to produce plants with knockouts of targeted genes: successful gene knockouts using artificial nucleases such as transcription activator-like effector nucleases (TALENs) and CRISPR/Cas have already been reported in rice (Li et al., 2012; Feng et al., 2013; Jiang et al., 2013; Miao et al., 2013; Shan et al., 2013; Xu et al., 2014). A series of mutants in which genes involved in the NHEJ pathway are disrupted can now be produced easily in the same variety. Moreover, targeted mutagenesis using artificial nucleases enables “null” and “truncated” mutants to be produced as necessary, unlike conventional mutagenesis approaches and T-DNA tagging lines. These new technologies could help resolve some of the conflicting results in this field. For example, the involvement in stable transformation of the *Arabidopsis lig4* mutant described above remains controversial, and conflicting results have also been reported in mutants in pathways other than NHEJ. For example, the plant protein VirE2 binding protein 1 (VIP1) has long been considered important in transient and/or stable transformation (Tzfira et al., 2001, 2002; Li et al., 2005a), but results that dispute this view have been reported recently (Shi et al., 2014). A study using knockout mutants of *Lig4* and *Vip1* in rice produced by artificial nucleases could lead to an answer.

Gene targeting (GT) is a transformation technology that can modify a targeted gene in a predicted manner. Successful examples of gene modification via GT have been reported in several higher plants including rice and *Arabidopsis*. However, rice is currently the only flowering plant in which target modification via GT can be performed routinely (Endo et al., 2007; Saika et al., 2011a; Nishizawa-Yokoi et al., 2014; Osakabe et al., 2014). In particular, GT using positive-negative selection enables the introduction of desirable mutations that cause amino acid substitutions in the targeted rice gene (Wakasa et al., 2012; Dang et al., 2013; Nishizawa-Yokoi et al., 2014). Molecular analyses and structure-based protein engineering can reveal the essential amino acids involved in protein–protein interactions. For example, as mentioned above, *Agrobacterium* virE2 protein interacts with Xrcc4 proteins (Vaghchhipawala et al., 2012) It is easy to produce mutant rice plants in which mutated Xrcc4 proteins are expressed. The comparison of transient/stable transformation frequencies in *xrcc4* mutant rice lines deficient in the interaction with virE2 protein or the enhancement of Lig4 activity will be able to provide clues as to the exact roles of Xrcc4 protein in stable transformation. Similarly, a set of rice mutants expressing mutated proteins that have lost the ability to interact with proteins derived from *Agrobacterium* will be useful in analyzing transformation frequency. Such studies will offer new insights into the stable transformation process. Moreover, further analysis will enable the discovery of chemicals effective in strictly activating or suppressing stable transformation, and these could be applied not only in experimental procedures in plants that are recalcitrant to *Agrobacterium*-mediated transformation but also in cases where T-DNA integration is not desired, such as site-directed mutagenesis using artificial nucleases and GT.

## Conflict of Interest Statement

The authors declare that the research was conducted in the absence of any commercial or financial relationships that could be construed as a potential conflict of interest.

## References

[B1] Adachi S. Minamisawa K. Okushima Y. Inagaki S. Yoshiyama K. Kondou Y. (2011). Programmed induction of endoreduplication by DNA double-strand breaks in *Arabidopsis*. *Proc. Natl. Acad. Sci. U.S.A.* 108 10004–10009. 10.1073/pnas.1103584108 21613568 PMC3116379

[B2] Anand A. Krichevsky A. Schomack S. Lahaye T. Tzfira T. Tang Y. H. (2007). *Arabidopsis* VIRE2 INTERACTING PROTEIN2 is required for *Agrobacterium* T-DNA integration in plants. *Plant Cell* 19 1695–1708. 10.1105/tpc.106.042903 17496122 PMC1913729

[B3] Bundock P. Den Dulk-Ras A. Beijersbergen A. Hooykaas P. J. (1995). Trans-kingdom T-DNA transfer from *Agrobacterium tumefaciens* to *Saccharomyces cerevisiae*. *EMBO J.* 14 3206–3214 7621833 10.1002/j.1460-2075.1995.tb07323.x PMC394382

[B4] Charbonnel C. Allain E. Gallego M. E. White C. I. (2011). Kinetic analysis of DNA double-strand break repair pathways in *Arabidopsis*. *DNA Repair* 10 611–619. 10.1016/j.dnarep.2011.04.002 21530420

[B5] Dang T. T. Shimatani Z. Kawano Y. Terada R. Shimamoto K. (2013). Gene editing a constitutively active OsRac1 by homologous recombination-based gene targeting induces immune responses in rice. *Plant Cell Physiol.* 54 2058–2070. 10.1093/pcp/pct147 24158358

[B6] Endo M. Nakayama S. Umeda-Hara C. Ohtsuki N. Saika H. Umeda M. (2012). CDKB2 is involved in mitosis and DNA damage response in rice. *Plant J.* 69 967–977. 10.1111/j.1365-313X.2011.04847.x 22092531 PMC3440594

[B7] Endo M. Osakabe K. Ono K. Handa H. Shimizu T. Toki S. (2007). Molecular breeding of a novel herbicide-tolerant rice by gene targeting. *Plant J.* 52 157–166. 10.1111/j.1365-313X.2007.03230.x 17883686

[B8] Feng Z. Zhang B. Ding W. Liu X. Yang D.-L. Wei P. (2013). Efficient genome editing in plants using a CRISPR/Cas system. *Cell Res.* 23 1229–1232. 10.1038/cr.2013.114 23958582 PMC3790235

[B9] Friesner J. Britt A. B. (2003). Ku80- and DNA ligase IV-deficient plants are sensitive to ionizing radiation and defective in T-DNA integration. *Plant J.* 34 427–440. 10.1046/j.1365-313X.2003.01738.x 12753583

[B10] Gallego M. E. Bleuyard J. Y. Daoudal-Cotterell S. Jallut N. White C. I. (2003). Ku80 plays a role in non-homologous recombination but is not required for T-DNA integration in *Arabidopsis*. *Plant J.* 35 557–565. 10.1046/j.1365-313X.2003.01827.x 12940949

[B11] Gelvin S. B. (2010). Plant proteins involved in *Agrobacterium*-mediated genetic transformation. *Annu. Rev. Phytopathol.* 48 45–68. 10.1146/annurev-phyto-080508-081852 20337518

[B12] Gheysen G. Villarroel R. Van Montagu M. (1991). Illegitimate recombination in plants: a model for T-DNA integration. *Genes Dev.* 5 287–297. 10.1101/gad.5.2.287 1995418

[B13] Hiei Y. Komari T. (2008). *Agrobacterium*-mediated transformation of rice using immature embryos or calli induced from mature seed. *Nat. Protoc.* 3 824–834. 10.1038/nprot.2008.46 18451790

[B14] Hiei Y. Ohta S. Komari T. Kumashiro T. (1994). Efficient transformation of rice (Oryza sativa L.) mediated by *Agrobacterium* and sequence analysis of the boundaries of the T-DNA. *Plant J.* 6 271–282. 10.1046/j.1365-313X.1994.6020271.x 7920717

[B15] Hong J.-P. Byun M. Y. An K. Yang S.-J. An G. Kim W. T. (2010). OsKu70 is associated with developmental growth and genome stability in rice. *Plant Physiol.* 152 374–387. 10.1104/pp.109.150391 19923234 PMC2799371

[B16] Jia Q. Bundock P. Hooykaas P. J. J. Pater S. D. (2012). *Agrobacterium tumefaciens* T-DNA integration and gene targeting in *Arabidopsis thaliana* non-homologous end-joining mutants. *J. Bot.* 2012 13. 10.1155/2012/989272

[B17] Jiang W. Zhou H. Bi H. Fromm M. Yang B. Weeks D. P. (2013). Demonstration of CRISPR/Cas9/sgRNA-mediated targeted gene modification in *Arabidopsis*, tobacco, sorghum and rice. *Nucleic Acids Res.* 41 e188. 10.1093/nar/gkt780 PMC3814374 23999092

[B18] Kimura S. Sakaguchi K. (2006). DNA repair in plants. *Chem. Rev.* 106 753–766. 10.1021/cr040482n 16464023

[B19] Kunik T. Tzfira T. Kapulnik Y. Gafni Y. Dingwall C. Citovsky V. (2001). Genetic transformation of HeLa cells by *Agrobacterium*. *Proc. Natl. Acad. Sci. U.S.A.* 98 1871–1876. 10.1073/pnas.041327598 11172043 PMC29349

[B20] Lacroix B. Citovsky V. (2013). The roles of bacterial and host plant factors in *Agrobacterium*-mediated genetic transformation. *Int. J. Dev. Biol.* 57 467–481. 10.1387/ijdb.130199b1 24166430 PMC9478875

[B21] Li J. X. Krichevsky A. Vaidya M. Tzfira T. Citovsky V. (2005a). Uncoupling of the functions of the *Arabidopsis* VIP1 protein in transient and stable plant genetic transformation by *Agrobacterium*. *Proc. Natl. Acad. Sci. U.S.A.* 102 5733–5738. 10.1073/pnas.0404118102 15824315 PMC556277

[B22] Li J. X. Vaidya M. White C. Vainstein A. Citovsky V. Tzfira T. (2005b). Involvement of KU80 in T-DNA integration in plant cells. *Proc. Natl. Acad. Sci. U.S.A.* 102 19231–19236. 10.1073/pnas.0506437103 16380432 PMC1323163

[B23] Li T. Liu B. Spalding M. H. Weeks D. P. Yang B. (2012). High-efficiency TALEN-based gene editing produces disease-resistant rice. *Nat. Biotechnol.* 30 390–392. 10.1038/nbt.2199 22565958

[B24] Liang Z. B. Tzfira T. (2013). In vivo formation of double-stranded T-DNA molecules by T-strand priming. *Nat. Commun.* 4 2253. 10.1038/ncomms3253 23963047

[B25] Magori S. Citovsky V. (2011). Epigenetic control of *Agrobacterium* T-DNA integration. *Biochim. Biophys. Acta Gene* 1809 388–394. 10.1016/j.bbagrm.2011.01.007 PMC3108046 21296691

[B26] Mayerhofer R. Koncz-Kalman Z. Nawrath C. Bakkeren G. Crameri A. Angelis K. (1991). T-DNA integration: a mode of illegitimate recombination in plants. *EMBO J.* 10 697–704 2001683 10.1002/j.1460-2075.1991.tb07999.x PMC452704

[B27] Mestiri I. Norre F. Gallego M. E. White C. I. (2014). Multiple host-cell recombination pathways act in *Agrobacterium*-mediated transformation of plant cells. *Plant J.* 77 511–520. 10.1111/tpj.12398 24299074

[B28] Miao J. Guo D. Zhang J. Huang Q. Qin G. Zhang X. (2013). Targeted mutagenesis in rice using CRISPR-Cas system. *Cell Res.* 23 1233–1236. 10.1038/cr.2013.123 23999856 PMC3790239

[B29] Mysore K. S. Bassuner B. Deng X. B. Darbinian N. S. Motchoulski A. Ream W. (1998). Role of the *Agrobacterium tumefaciens* VirD2 protein in T-DNA transfer and integration. *Mol. Plant Microbe Interact.* 11 668–683. 10.1094/mpmi.1998.11.7.668 9650299

[B30] Nam J. Matthysse A. G. Gelvin S. B. (1997). Differences in susceptibility of *Arabidopsis* ecotypes to crown gall disease may result from a deficiency in T-DNA integration. *Plant Cell* 9 317–333. 10.1105/tpc.9.3.317 9090878 PMC156921

[B31] Nishimura A. Aichi I. Matsuoka M. (2006). A protocol for *Agrobacterium*-mediated transformation in rice. *Nat. Protoc.* 1 2796–2802. 10.1038/nprot.2006.469 17406537

[B32] Nishimura A. Ashikari M. Lin S. Takashi T. Angeles E. R. Yamamoto T. (2005). Isolation of a rice regeneration quantitative trait loci gene and its application to transformation systems. *Proc. Natl. Acad. Sci. U.S.A.* 102 11940–11944. 10.1073/pnas.0504220102 16091467 PMC1187985

[B33] Nishizawa-Yokoi A. Endo M. Ohtsuki N. Saika H. Toki S. (2014). Precision genome editing in plants via gene targeting and piggyBac-mediated marker excision. *Plant J.* 10.1111/tpj.12693 [Epub ahead of print]. PMC4309413 25284193

[B34] Nishizawa-Yokoi A. Nonaka S. Saika H. Kwon Y. I. Osakabe K. Toki S. (2012). Suppression of Ku70/80 or Lig4 leads to decreased stable transformation and enhanced homologous recombination in rice. *New Phytol.* 196 1048–1059. 10.1111/j.1469-8137.2012.04350.x 23050791 PMC3532656

[B35] Osakabe K. Nishizawa-Yokoi A. Ohtsuki N. Osakabe Y. Toki S. (2014). A mutated cytosine deaminase gene, codA (D314A), as an efficient negative selection marker for gene targeting in rice. *Plant Cell Physiol.* 55 658–665. 10.1093/pcp/pct183 24371307

[B36] Ozawa K. Kawahigashi H. (2006). Positional cloning of the nitrite reductase gene associated with good growth and regeneration ability of calli and establishment of a new selection system for *Agrobacterium*-mediated transformation in rice (*Oryza sativa* L.). *Plant Sci.* 170 384–393. 10.1016/j.plantsci.2005.09.015

[B37] Pitzschke A. Hirt H. (2010). New insights into an old story: *Agrobacterium*-induced tumour formation in plants by plant transformation. *EMBO J.* 29 1021–1032. 10.1038/emboj.2010.8 20150897 PMC2845280

[B38] Richael C. M. Kalyaeva M. Chretien R. C. Yan H. Adimulam S. Stivison A. (2008). Cytokinin vectors mediate marker-free and backbone-free plant transformation. *Transgenic Res.* 17 905–917. 10.1007/s11248-008-9175-6 18320338

[B39] Riha K. Watson J. M. Parkey J. Shippen D. E. (2002). Telomere length deregulation and enhanced sensitivity to genotoxic stress in *Arabidopsis* mutants deficient in Ku70. *EMBO J.* 21 2819–2826. 10.1093/emboj/21.11.2819 12032094 PMC126030

[B40] Saika H. Nonaka S. Osakabe K. Toki S. (2012). Sequential monitoring of transgene expression following *Agrobacterium*-mediated transformation of rice. *Plant Cell Physiol.* 53 1974–1983. 10.1093/pcp/pcs135 23026817

[B41] Saika H. Oikawa A. Matsuda F. Onodera H. Saito K. Toki S. (2011a). Application of gene targeting to designed mutation breeding of high-tryptophan rice. *Plant Physiol.* 156 1269–1277. 10.1104/pp.111.175778 21543727 PMC3135912

[B42] Saika H. Sakamoto W. Maekawa M. Toki S. (2011b). Highly efficient visual selection of transgenic rice plants using green fluorescent protein or anthocyanin synthetic genes. *Plant Biotechnol.* 28 107–110. 10.5511/plantbiotechnology.10.1104a

[B43] Saika H. Toki S. (2009). Visual selection allows immediate identification of transgenic rice calli efficiently accumulating transgene products. *Plant Cell Rep.* 28 619–626. 10.1007/s00299-009-0671-9 19198844

[B44] Saika H. Toki S. (2010). Mature seed-derived callus of the model indica rice variety Kasalath is highly competent in *Agrobacterium*-mediated transformation. *Plant Cell Rep.* 29 1351–1364. 10.1007/s00299-010-0921-x 20853107 PMC2978894

[B45] Shan Q. Wang Y. Li J. Zhang Y. Chen K. Liang Z. (2013). Targeted genome modification of crop plants using a CRISPR-Cas system. *Nat. Biotechnol.* 31 686–688. 10.1038/nbt.2650 23929338

[B46] Shi Y. Lee L. Y. Gelvin S. B. (2014). Is VIP1 important for *Agrobacterium*-mediated transformation? *Plant J.* 9 848–860. 10.1111/tpj.12596 24953893

[B47] Shiboleth Y. Tzfira T. (2012). “*Agrobacterium*-mediated plant genetic transformation,” in *Plant Biotechnology and Agriculture: Prospects for the 21st Century,* eds Altman A. Hasegawa P. M. (Waltham, MA: Academic Press), 99–116. 10.1016/B978-0-12-381466-1.00007-9

[B48] Toki S. Hara N. Ono K. Onodera H. Tagiri A. Oka S. (2006). Early infection of scutellum tissue with *Agrobacterium* allows high-speed transformation of rice. *Plant J.* 47 969–976. 10.1111/j.1365-313X.2006.02836.x 16961734

[B49] Tzfira T. Vaidya M. Citovsky V. (2001). VIP1, an *Arabidopsis* protein that interacts with *Agrobacterium* VirE2, is involved in VirE2 nuclear import and *Agrobacterium* infectivity. *EMBO J.* 20 3596–3607. 10.1093/emboj/20.13.3596 11432846 PMC125502

[B50] Tzfira T. Vaidya M. Citovsky V. (2002). Increasing plant susceptibility to *Agrobacterium* infection by overexpression of the *Arabidopsis* nuclear protein VIP1. *Proc. Natl. Acad. Sci. U.S.A.* 99 10435–10440. 10.1073/pnas.162304099 12124400 PMC124932

[B51] Vaghchhipawala Z. E. Vasudevan B. Lee S. Morsy M. R. Mysore K. S. (2012). *Agrobacterium* may delay plant nonhomologous end-joining DNA repair via XRCC4 to favor T-DNA integration. *Plant Cell* 24 4110–4123. 10.1105/tpc.112.100495 23064322 PMC3517239

[B52] van Attikum H. Bundock P. Hooykaas P. J. J. (2001). Non-homologous end-joining proteins are required for *Agrobacterium* T-DNA integration. *EMBO J.* 20 6550–6558. 10.1093/emboj/20.22.6550 11707425 PMC125718

[B53] van Attikum H. Bundock P. Overmeer R. M. Lee L. Y. Gelvin S. B. Hooykaas P. J. J. (2003). The *Arabidopsis* AtLIG4 gene is required for the repair of DNA damage, but not for the integration of *Agrobacterium* T-DNA. *Nucleic Acids Res.* 31 4247–4255. 10.1093/nar/gkg458 12853643 PMC165973

[B54] van Attikum H. Hooykaas P. J. J. (2003). Genetic requirements for the targeted integration of *Agrobacterium* T-DNA in *Saccharomyces cerevisiae*. *Nucleic Acids Res.* 31 826–832. 10.1093/nar/gkg183 12560477 PMC149203

[B55] Wakasa Y. Hayashi S. Ozawa K. Takaiwa F. (2012). Multiple roles of the ER stress sensor IRE1 demonstrated by gene targeting in rice. *Sci. Rep.* 2 944. 10.1038/srep00944 PMC3517978 23230509

[B56] West C. E. Waterworth W. M. Jiang Q. Bray C. M. (2000). *Arabidopsis* DNA ligase IV is induced by γ-irradiation and interacts with an *Arabidopsis* homologue of the double strand break repair protein XRCC4. *Plant J.* 24 67–78. 10.1046/j.1365-313x.2000.00856.x 11029705

[B57] Xu R. Li H. Qin R. Wang L. Li L. Wei P. (2014). Gene targeting using the *Agrobacterium tumefaciens*-mediated CRISPR-Cas system in rice. *Rice* 7 5. 10.1186/s12284-014-0005-6 PMC4052633 24920971

